# Preoperatively administered single dose of dexketoprofen decreases pain intensity on the first 5 days after craniotomy: A single-centre placebo-controlled, randomized trial

**DOI:** 10.1515/tnsci-2022-0323

**Published:** 2023-12-16

**Authors:** Éva Simon, Csaba Csipkés, Dániel Andráskó, Veronika Kovács, Zoltán Szabó-Maák, Béla Tankó, Gyula Buchholcz, Béla Fülesdi, Csilla Molnár

**Affiliations:** Department of Anesthesiology and Intensive Care, Faculty of Medicine, University of Debrecen, Debrecen, Hungary; University Pharmacy, Clinical Centre, University of Debrecen, Debrecen, Hungary

**Keywords:** postcraniotomy headache, preemptive analgesia, dexketoprofen

## Abstract

**Background and purpose:**

Headache attributed to craniotomy is an underestimated and under-treated condition. Previous studies confirmed the efficacy of preemptive analgesia with non-steroidal anti-inflammatory agents. The aim of the present work was to test the hypothesis of whether a single preoperatively administered dose of dexketoprofen (DEX) has the potency to decrease postcraniotomy headache (PCH) as compared to placebo (PL).

**Patients and methods:**

This is a single-centre, randomized, PL-controlled trial comparing the effect of a single oral dose of 25 mg DEX to PL on the intensity of PCH. Patients undergoing craniotomy were randomly allocated to DEX and PL groups. Patients rated their actual and worst daily pain using visual analogue scale (VAS) scores during intrahospital treatment (0–5 days) and 30 and 90 days postoperatively.

**Results:**

Two hundred patients were included. DEX decreased the worst daily pain intensity in the first 24 h only; the 5-days cumulative score of actual pain was 9.7 ± 7.9 cm for the DEX group and 12.6 ± 10.5 cm for the PL group, respectively (*p* = 0.03). This difference disappeared in the late, 30-, and 90-day follow-up period. No differences in VAS scores could be detected in supra- and infratentorial cases among the DEX and PL groups.

**Conclusions:**

A single preoperative dose of 25 mg of DEX slightly decreases the intensity of PCH in the first 5 days after craniotomy but it does not have an effect on chronic headaches and postoperative analgesic requirements.

Postcraniotomy headache (PCH, according to the more recent term: “headache attributed to craniotomy”) is a secondary headache that develops within 7 days after surgery and is most serious at the site of the intervention. It begins usually within the first few hours–days after craniotomy and resolves within the acute postoperative period in the majority of the cases [1]. The pain may persist for over 3 months in up to 30% of the cases [2,3], corresponding to the term persistent headache attributed to craniotomy [1]. The intensity of pain is usually moderate to severe (visual analogue scale (VAS) score: \[\ge ]\] 4 cm); its incidence peaks usually at 12 h after surgery (60–79% of the patients are affected) and its severity is highest within 48 h after the surgical intervention, showing a declining tendency thereafter [4,5].

PCH is a multifactorially determined postoperative condition. Among others, the age and gender of the patients and the localization of the surgical procedure may influence its appearance, severity, and tendency to develop a persistent form [6]. The most important aim of all therapeutic interventions is to relieve postoperative pain and the avoidance or reduction of the use of opioid analgesics. Accordingly, a multimodal analgesic strategy is proposed to decrease the intensity of PCH in the postoperative setting. Besides the administration of analgesics during the intraoperative and the postoperative period, preoperative administration of non-steroidal anti-inflammatory agents (preemptive analgesia) as well as local infiltration of the surgical field before craniotomy were documented to reduce postoperative pain and analgesic requirements [6,7]. In a previous randomized study, it was found that diclofenac in a single preoperatively administered dose reduces the intensity of acute PCH and decreases analgesic requirements in the postoperative setting [5]. However, the use of diclofenac, as a non-selective COX inhibitor raises several concerns, such as the risk of gastrointestinal bleeding and cardiovascular complications and thus there is a tendency toward using selective agents with fewer side effects [8]. In this respect, in the present randomized trial, we attempted to test the hypothesis of whether a single preoperatively administered dose of dexketoprofen (DEX) has the potency to decrease PCH as compared to placebo (PL).

## 1 Patients and methods

We enrolled consecutive adults having elective craniotomies for brain tumors at the Department of Neurosurgery, University of Debrecen. Only patients who were alert preoperatively were included. Patients with chronic headaches in the history, taking non-steroidal anti-inflammatory agents prior to surgery, and with preoperative aphasia were excluded.

Before starting the study, we performed a sample size calculation using the clincal.com. In a previous pilot study [9], the cumulative VAS score for the first 5 postoperative days was found to be 14.4 ± 9 cm. Anticipating a 30% decrease in the cumulative VAS scores after a single dose of preoperatively administered DEX and calculating with an alpha of 5% and with a power of 90% resulted in 198 patients (99 in PL and 99 in DEX groups) being included. We finally decided to include 200 patients to account for eventual dropouts and technical failures during the study and follow-up.

Using a random generator, we prepared sequentially numbered opaque envelopes to ensure the allocation of the patients to treatment and PL groups. The envelopes were opened 1 h prior to the anticipated start of surgery by a participant who was not involved in the anesthesia and the follow-up of the certain patient. This physician administered either 25 mg DEX (Ketodex 25 mg tablets, Berlin-Chemie Menarini, Budapest, Hungary) or PL (produced by the local pharmacy) to the patient to take it orally with a sip of water, according to the randomization. This double blinding ensured that neither the intraoperative anesthetist nor the anesthetist performing the VAS score reports and follow-up was aware of patient grouping.

Anesthesia was induced with propofol (1–2.5 mg/kg) and subsequently maintained with fentanyl, rocuronium, and sevoflurane. Fentanyl was given as an initial bolus of 100–150 μg, followed by an infusion of 2 μg/kg/h. Before the surgical intervention, the surgical field was infiltrated by the neurosurgeon with a combination of 2% lidocaine and epinephrine.

In the postoperative phase, patients were transferred to the neurosurgical HDU. A stepwise, protocolized analgesic regime was followed. Severity of the pain was regularly assessed by the ICU nurses and independent physicians who were not aware of the patient grouping status (DEX or PL). The 0–10 VAS scores was used for the subjective pain rating of the patients on the day of surgery and every day during the first five postoperative days. The patients had to rate their actual pain at the time of assessment and the worst pain that they experienced during the last 24 h. Additional analgesics were given in all cases when the reported worst pain severity exceeded VAS 3 cm. Per protocol, the initial treatment was 1 g intravenous paracetamol, followed by oral tramadol at a dose of 100 mg, in case the paracetamol was ineffective. Morphine was reserved for patients in whom VAS scores remained >3 cm after paracetamol and tramadol administration. The daily cumulative dose of analgesics was registered in the first 5 postoperative days. Cumulative analgetic doses during the first 5 postoperative days were compared among the groups. After the patients were released from the hospital, they were asked to rate their actual pain experience during their daily life on the 30th and 90th postoperative days using phone calls by an independent study team member.

A normality test revealed that VAS scores and analgesic requirements were not normally distributed; Mann–Whitney *U* tests were thus used for comparisons. Repeated-measure analysis of variance was used for multiple comparisons. Results are presented as mean and 95% confidence intervals or mean and standard deviations as appropriate; *P* < 0.05 was considered statistically significant.


**Ethical approval:** The research related to human use complied with all the relevant national regulations, and institutional policies and in accordance with the tenets of the Helsinki Declaration, and has been approved by the authors’ institutional review board or equivalent committee. This study was conducted with approval from the University of Debrecen Medical Ethics Committee (registration number: DE RKEB/IKEB: 4669-2016, responsible person: József Szentmiklósi; Department of Pharmacology, University of Debrecen, 98, Nagyerdei krt., Debrecen, Hungary, Phone: +36 52411600). The study was registered in EudraCT (registration number: 2017-000414-35, principal investigator: Csilla Molnár).
**Informed consent:** Informed consent has been obtained from all individuals included in this study.

## 2 Results

**Figure 1:**
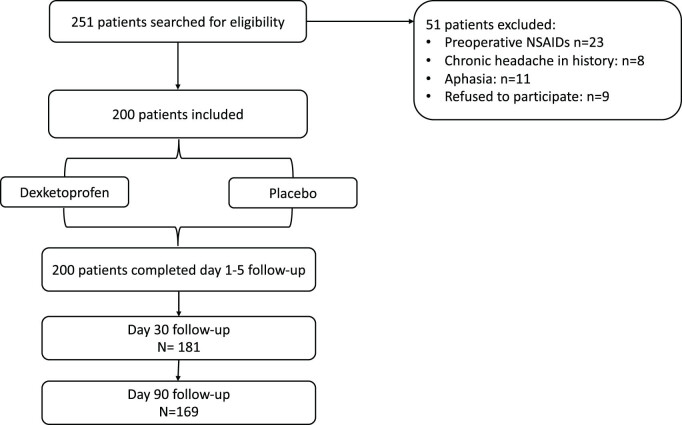
Patient selection and randomization (consort diagram).

Consecutive patients selected for elective craniotomies at the Department of Neurosurgery, University of Debrecen, were searched for eligibility during routine preoperative anesthesiology consultations between the period of June 1, 2017, and December 2019. The details of patient selection, exclusion, randomization, and follow-up are depicted in Figure 1, and the clinical characteristics of the included patients are summarized in Table 1.

**Table 1:** Clinical characteristics of patients

Age (years)	52.3 ± 14.9
Gender (F/M)	108/92
Preoperative headache (Y/N)	42/158
Location of surgery (supratentorial/infratentorial)	141/39
**Histology of the lesion operated**	
Meningeoma	60
Metastatic tumor	53
Glioblastoma	30
Astrocytoma	16
Oligodendroglioma	12
Cavernoma	5
Craniopharyngeoma	3
Plexus papilloma	2
Ependymoma	2
Acoustic neurinoma	3
Arteriovenous malformation	1
Other	12
Duration of surgery (min)	222.0 ± 71.2

### 2.1 Comparison of postoperative pain intensities on days 0–5 in the DEX and the PL groups:

**Figure 2:**
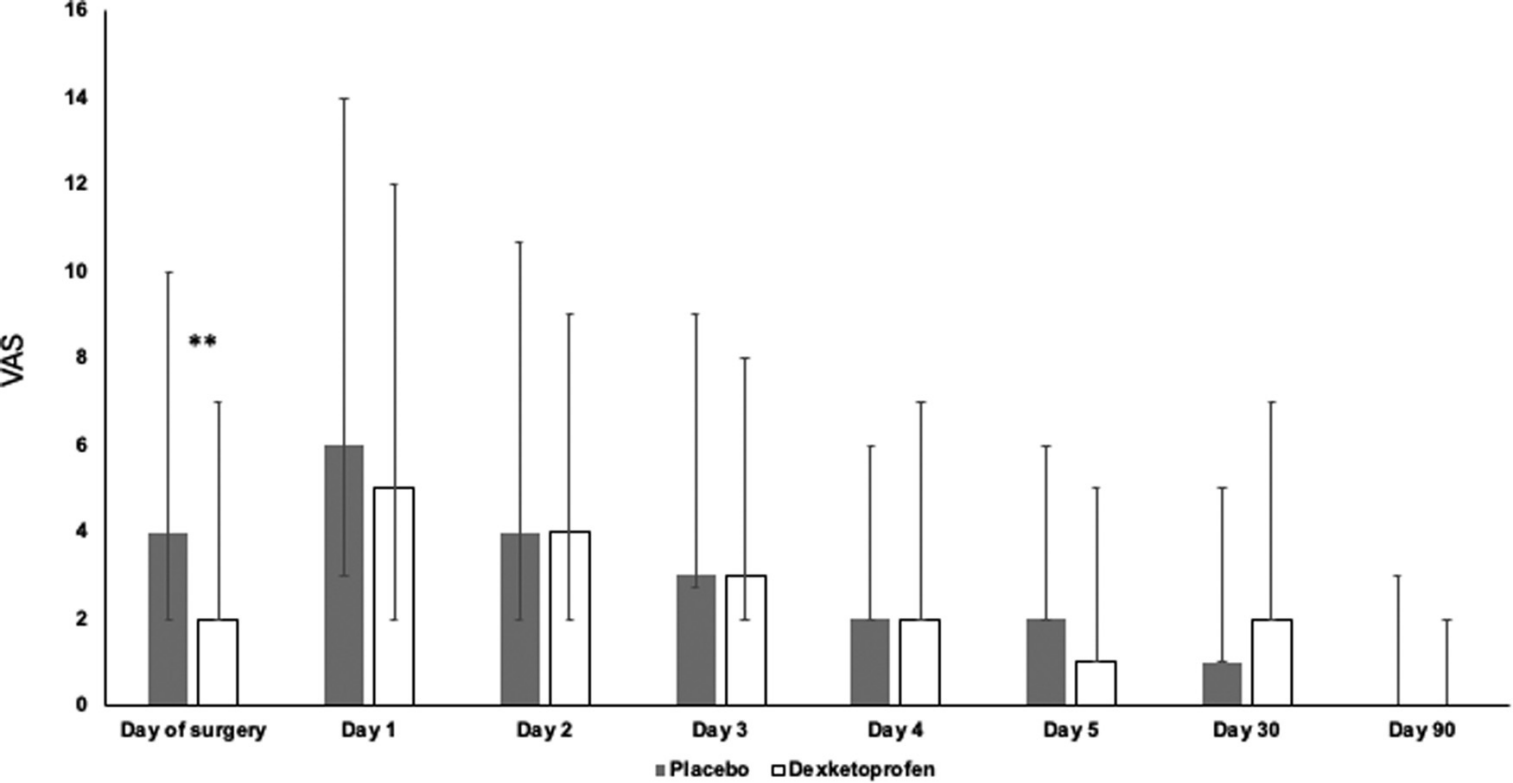
VAS in the DEX and PL groups. Medians and 25–75% interquartile ranges are shown. ** indicates a statistical difference of *p* < 0.01.

Preoperative headache was reported in 42 patients preoperatively, and the cases were equally distributed in the treatment and the PL group (DEX: 22 cases; PL: 20 cases). There were no differences between preoperatively reported pain intensities among the two groups. Preoperatively administered DEX resulted in a lower VAS score on the day of surgery: PL group 4 (2–6) cm vs DEX group 2 (0–5) cm, respectively (*P* < 0.01). This difference disappeared in the following 5 postoperative days and the similarity in therapeutic efficacy between DEX and PL persisted for days 30 and 90 (Figure 2).

After computing the cumulative actual VAS scores reported by the patients at the regular check of headache, the 5-day cumulative score was 9.7 ± 7.9 cm for the DEX group and 12.6 ± 10.5 cm for the PL group (*p* = 0.03). However, when the cumulative value of the worst VAS scores for the first 5 postoperative days was computed, there were no statistically significant differences in the pain intensity between the groups (DEX: 6 (5–8) cm vs PL: 7 (5–8) cm, *p* = 0.051).

We also evaluated the treatment effect of DEX by forming different VAS subgroups (no pain, mild, moderate, and severe pain). The results are summarized in Table 2 for worst daily VAS scores and in Table 3 for actual VAS scores. The preoperatively administered DEX treatment was found to be effective on the day of surgery and the first postoperative day as compared to PL. It is also worth mentioning that roughly one-third of the patients at 30 days, and only roughly 50% at 90 days after craniotomy reported having no pain and the majority still experienced a mild or moderate headache in both treatment arms, necessitating analgesic treatment.

**Table 2:** Comparison of VAS score categories between the DEX and PL arms according to the time after surgery

	Day 0	Day 1	Day 2	Day 3	Day 4	Day 5	Day 30	Day 90
**PL group**								
No pain	22	9	20	25	33	39	32	51
VAS 1–3	24	22	20	29	27	32	32	18
VAS 4–6	31	28	34	28	28	18	15	15
VAS 7–10	23	41	26	18	12	12	12	2
**DEX group**								
No pain	31	4	18	16	29	38	37	55
VAS 1–3	33	30	25	40	38	33	31	20
VAS 4–6	28	39	41	31	26	21	19	10
VAS 7–10	8	27	16	13	7	9	5	2
Chi^2^	10.4	7.8	3.7	4.7	3.5	0.68	3.7	1.25
*p*-value	0.01	<0.05	0.29	0.19	0.3	0.87	0.29	0.74

The number of cases is presented in the table. Note that in the PL group, 91 and 86 patients, whereas in the DEX group, 92 and 87 patients could be reached on days 30 and 90, respectively.

**Table 3:** Comparison of actual VAS score categories between the DEX and PL arms according to the time after surgery

	Day 0	Day 1	Day 2	Day 3	Day 4	Day 5	Day 30	Day 90
**PL group**								
No pain	23	35	43	49	51	59	32	51
VAS 1–3	23	35	29	31	32	27	32	18
VAS 4–6	31	23	18	16	9	10	15	15
VAS 7–10	23	7	10	4	8	4	12	2
**DEX group**								
No pain	31	30	43	50	62	64	37	55
VAS 1–3	33	43	43	35	23	24	31	20
VAS 4–6	28	22	9	12	11	9	19	10
VAS 7–10	8	5	5	3	4	3	5	2
Chi^2^	10.4	1.56	7.4	0.96	4.1	0.57	3.7	1.25
*p*-value	0.01	0.66	0.06	0.8	0.25	0.9	0.29	0.74

The number of cases is presented in the table. Note, that in the PL group, 91 and 86 patients, whereas in the DEX group, 92 and 87 patients could be reached on days 30 and 90, respectively.

### 2.2 Comparison of postoperative pain intensities in supratentorial and infratentorial surgeries

**Figure 3:**
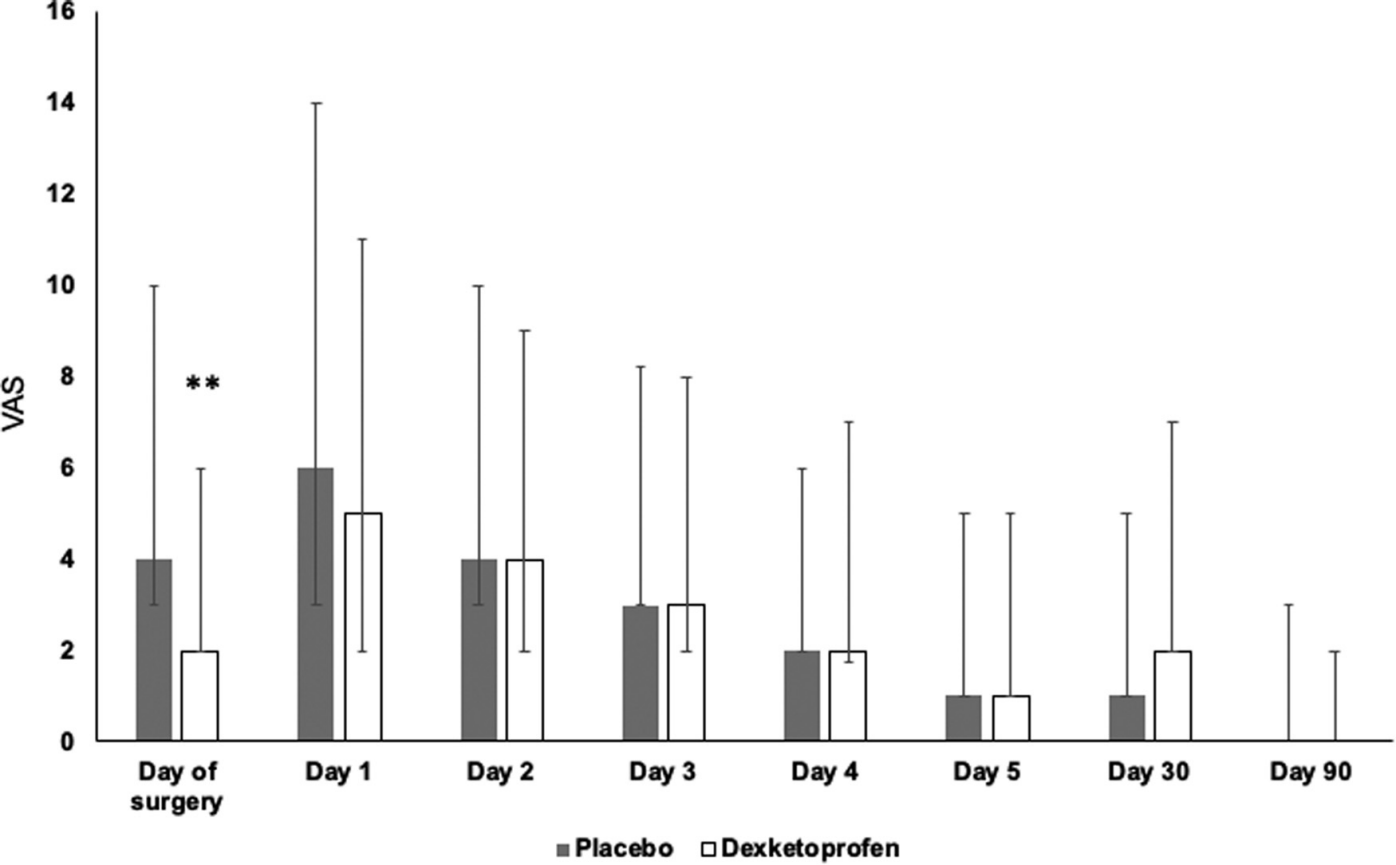
VAS in the DEX and PL groups in patients with supratentorial surgeries. Medians and 25–75% interquartile ranges are shown. ** indicates a statistical difference of *p* < 0.01.

**Figure 4:**
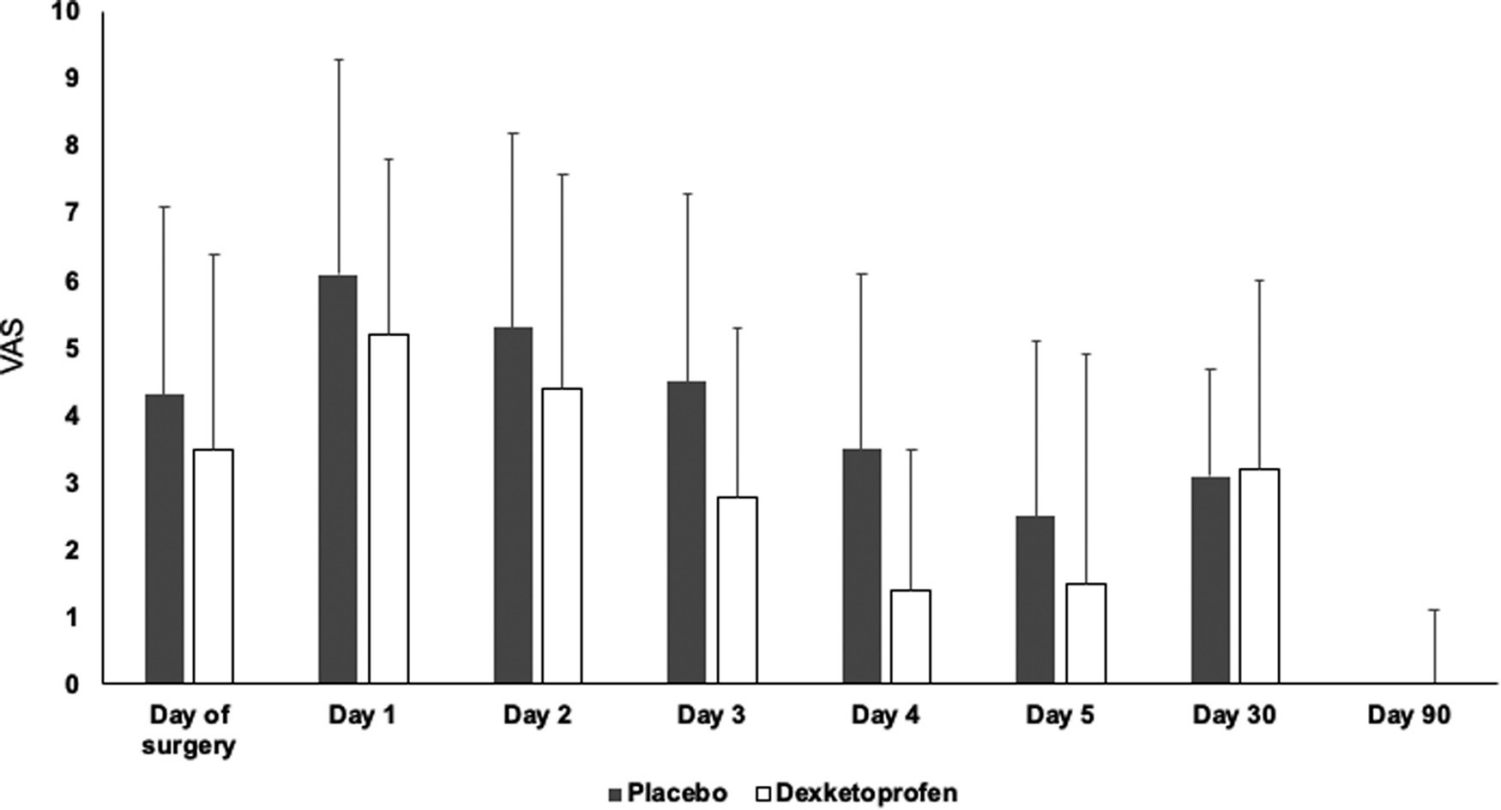
VAS in the DEX and PL groups in patients with infratentorial surgeries. Mean and standard deviations are shown.

Postoperative VAS scores in patients who underwent supratentorial craniotomies were lower in the DEX group (*n* = 83) as compared to PL (*n* = 77) on the day of surgery but not in the following 5 postoperative days (Figure 3). However, a single dose of DEX was not proven to be effective in any early postoperative periods in infratentorial interventions (Figure 4).

### 2.3 Comparison of cumulative doses of analgesics in the DEX and PL groups

No additional opioid was given in any groups in the postoperative period. The analgesic requirements were similar in the two groups during the intrahospital phase.

## 3 Discussion

In the present PL-controlled trial, we tested the efficacy of a single dose of DEX compared to PL in decreasing pain intensity in the postoperative phase and we also checked whether the active treatment results in decreased postoperative analgesic requirements. It was found that a single 25 mg dose of DEX decreases headache mostly on the day after surgery and has a discrete analgesic effect between days 1 and 5. The analgesic requirements were similar in the two groups during the intrahospital phase. No differences could be found in the incidence of postcraniotomy pain at 30 and 90 days after surgery.

Headache attributed to craniotomy is an underestimated and under-treated condition that might be related to the nature of the surgical intervention (nerve damage, dural traction, nerve entrapments to the scar, muscle incision, and neuroma formation), but other factors, such as younger age, female sex, and preoperative personality may influence its appearance and severity [3,6,10]. A multimodal therapeutic approach is proposed that meets the requirements of having an effective anti-analgetic effect but lacks respiratory depression and deep sedation in the postoperative setting [11]. In a recent review, Bello and co-workers proposed combining different preventive techniques, such as scalp infiltration and preoperatively administered non-steroidal inflammatory agents with postoperative analgesics, including short-acting opioids and non-steroidal anti-inflammatory agents [6].

There are only a few studies that assessed the efficacy of non-steroidal anti-inflammatory agents after craniotomies [5,12,13,14,15,16,17], but in the vast majority of these studies, the initiation of therapy was in the postoperative phase. In a Cochrane review, Galvin and co-authors critically evaluated the possible pharmacological interventions that may prevent acute PCHs [7]. Again, in these studies, non-steroidal anti-inflammatory agents were administered either just before closure [12,13,16,17,18] or in the postoperative phase [14,15] but data on the preemptive analgesic effect of preoperatively administered NSAIDs are scarce. More importantly, in the majority of the institutions, no local protocols or international guidelines are available for prevention and treatment [19,20].

According to the concept of preemptive analgesia [21], preoperatively administered analgesics should ameliorate the mechanisms that are involved in the development of pain. As the initiation of pain starts with a neurogenic inflammation, an ideal agent should have both anti-inflammatory and anti-analgesic properties. In this line, some of the previous studies attempted to test the effectiveness of preoperatively administered NSAIDs on PCH. In a PL-controlled trial, Ryan studied the efficacy of a single preoperative dose of 50 mg rofecoxib and found no clinical benefit in terms of decreasing pain intensity and decreasing analgesic requirements [22]. It must be noted that most probably the study was underpowered to answer the question (19 patients and 15 patients finished it in the treatment and the PL arms, respectively). In another randomized study, Molnár and co-workers proved the efficacy of a single 100 mg dose of diclofenac during the first postoperative days [5]. VAS scores during the first 5 postoperative days were lower in patients with diclofenac pretreatment and this effect was more pronounced in the infratentorial surgical group. The authors also reported on a slight decrease in postoperative analgesic requirements in the diclofenac-treated group. In contrast, rofecoxib [21] and DEX did not exert a preventive effect. Thus, preoperative administration of NSAIDs for preemptive analgesia, in general, is not supported by previous clinical studies, including the present trial, and this may need to be explained.

In fact, different non-steroidal agents (diclofenac, parecoxib, DEX, methimazole, and ibuprofen) given before the closure of the operative field or right after the end of surgery were proven to be effective for the first 24 h [7] and this was also found in our study using DEX. The pharmacological effects of NSAIDs are due to the blocking of COX and the consequent reduction in the synthesis of prostaglandins, which leads to a decrease in inflammation, pain, and fever [8]. It is known that the anti-inflammatory effect of NSAIDs is mainly related to decreasing the prostaglandins responsible for vasodilation, especially PGE2 [23,24]. Diclofenac as a non-selective COX1 and COX2 inhibitor is known for its potent PGE2-decreasing potency. During the administration of diclofenac, it was found that roughly 40% of PGE2 inhibition may be responsible for analgesic efficacy (mostly related to increasing pain threshold at the peripheral nerve ending and decreasing central sensitization process) and the rest of the effect is an anti-inflammatory effect [20]. In contrast to diclofenac, DEX has more potent analgesic than anti-inflammatory effects, and several reports suggested that its analgesic activity is exerted to another mechanism that is not related to prostaglandin synthesis [25].

It is widely accepted that at the site of a tissue injury, a local inflammatory process plays an important role in the initiation of pain at the level of peripheral nerve endings [26] by being the main factor in transforming a mechanical injury into an electrical stimulus that can be forwarded to the dorsal horn. Based on these observations, it is conceivable that the combination of anti-inflammatory and analgesic effects may be responsible for the preemptive analgesic potency of some non-steroidal anti-inflammatory agents. According to this assumption, the anti-inflammatory effect may decrease the pain stimulus at the site of initiation, and the anti-analgesic effect is rather related to the other parts of the pain pathway. This concept is supported by the previous study of Molnár and co-workers, who found that preoperatively administered single-dose diclofenac more effectively reduced postoperative pain intensities in infratentorial surgeries where usually a more widespread tissue injury occurs [5].

Finally, we mention the limitations of our study. First, this was a single-centre trial and the postoperative analgesic treatment was performed according to the local protocols that may be different in other hospitals. We also may be criticized for the relatively low dose of DEX (25 mg). In fact, a 25 mg dose of DEX is just above the threshold analgesic dose of the drug [25], but it corresponds to an analgesic dose of 75 mg diclofenac [8] and we decided to administer this dose to keep the risk of possible side effects low along with ensuring its analgesic effect.

We conclude that although there might be some indications for the administration of NSAIDs for preemptive analgesia during craniotomies, a single dose of 25 mg DEX is marginally effective for this purpose. Non-steroidal agents given in a single dose preoperatively have been reported to be safe during craniotomies, but further studies, also taking the anti-inflammatory and analgesic properties and effective doses of the different NSAIDs into account, are needed to find the effective preemptive analgesic strategy.
